# Vaccine Hesitancy and Refusal: Behavioral Evidence from Rural Northern Nigeria

**DOI:** 10.3390/vaccines9091023

**Published:** 2021-09-14

**Authors:** Ryoko Sato, Yoshito Takasaki

**Affiliations:** 1 Center for Health Decision Science, Harvard T.H. Chan School of Public Health, 90 Smith Street, 332-1, Boston, MA 02120, USA; 2 Graduate School of Economics, University of Tokyo, 7-3-1 Hongo, Bunkyo-ku, Tokyo 1130033, Japan; takasaki@e.u-tokyo.ac.jp

**Keywords:** vaccine hesitancy, vaccine refusal, field experiment, northern Nigeria

## Abstract

It is widely believed that vaccine hesitancy is prevalent in African countries, although this belief is without rigorous evidence. Our field experiment in rural northern Nigeria behaviorally measured the prevalence of vaccine hesitancy—the non-uptake of vaccines despite their availability due to non-monetary factors directly associated with vaccination. We randomly assigned two tasks to women: answering a short survey at their house vs. additionally receiving a free tetanus vaccine by submitting a voucher. The differences in their completion rates captured vaccine hesitancy, showing the rate to be about 13%. Our study reveals that absolute refusers with negative willingness to pay (WTP) for vaccines, who are likely to have strong misperceptions or a distrust of vaccines, account for about half of vaccine hesitaters, while floating refusers with zero or weakly positive WTP, who are likely to be indifferent about vaccines, account for the other half. A simple intervention, such as a door-to-door vaccination campaign, is likely to be effective for floating refusers, while interventions for absolute refusers need to effectively change their misperceptions or distrust of vaccines.

## 1. Introduction

Despite the seemingly obvious benefits of vaccination to prevent diseases [1], noncompliance with recommended vaccination is a worldwide phenomenon. In the U.S., only 66% of children complete all the recommended vaccinations [2]. In developing countries worldwide, almost 20 million infants are estimated to have been unreached by routine immunization in 2016, of which 60% are concentrated in 10 countries, one of which is Nigeria—the site of our study [3]. There were 34,000 global neonatal tetanus deaths in 2015 [4], and up to 16% of these occurred in Nigeria, even though such deaths can be effectively prevented by tetanus toxoid vaccination, which we study here. Nigeria remains one of the 25 countries still reporting neonatal tetanus as a cause of infant mortality [5].

Past studies suggest various demand-side reasons for the persistent problems of low vaccination take-up, such as monetary barriers, i.e., transportation costs and opportunity costs to attend health clinics [6]; information barriers, i.e., lack of knowledge of disease, vaccines, and vaccine schedules [7,8]; and psychological barriers, i.e., beliefs and attitudes against vaccination [9] (see Rainey et al. (2011) for a systematic review of barriers to vaccination in developing countries [10]).

To describe psychological barriers to vaccination, the literature recently introduced an emerging term: vaccine hesitancy, which refers to a delay in acceptance, or refusal, of vaccination despite the availability of vaccination services [11]. In developed countries in which the immunization rate has already reached over 90% for most vaccines, vaccine hesitancy is considered to be one of the main factors responsible for increasing risks of outbreaks of vaccine-preventable diseases [12,13]. The recent outbreak of measles in the U.S., for example, is considered to be largely due to vaccine hesitancy [14].

Vaccine hesitancy is believed to be a critical issue in African countries as well. The Nigerian vaccination boycott in 2003 is a famous event that was precipitated by political leaders and that contributed to the creation of persistent vaccine hesitancy in the region. In three northern states, polio immunization campaigns were boycotted due to a suspicion about the safety of the vaccine: Islamic leaders propagated a suspicion to the public that polio vaccines could make women infertile or lead them to contract HIV [15], which resulted in the refusal of polio vaccines in the general population. The decreased take-up of the polio vaccine in northern Nigeria resulted in increased polio-virus transmission throughout the country [16] and then into 20 neighboring countries [17]. Similar refusals to participate in vaccination campaigns for polio and tetanus due to distrust have been observed across Africa, including in Nigeria [18,19,20]. These episodes have led to the presumption commonly being held by researchers that vaccine hesitancy is prevalent [10,21]. 

Behavioral evidence for the prevalence of vaccine hesitancy is scarce. This is because the majority of extant observational studies have measured vaccine hesitancy by asking respondents their reasons for non-uptake of vaccines [8,22]. Such an approach relying on subjective measures, however, might fail to measure the actual prevalence of vaccine hesitancy because respondents do not see consequences by providing any kind of response. They might also have multiple reasons for non-uptake, and their responses might not capture all the relevant reasons. 

In this paper, we consider vaccine hesitancy, among potential barriers to vaccination, as referring to the non-uptake of vaccines despite their availability due to non-monetary factors directly associated with vaccination. Examples of non-monetary factors include fear of needles, distrust of vaccine efficacy, concern about vaccine safety, and distrust of vaccine providers. Distinct from previous studies, our behavioral approach uses people’s actual vaccine uptake behaviors, which are consequential, as an outcome. Our field experiment was explicitly designed to measure vaccine hesitancy behaviorally by offering free tetanus vaccines on the doorstep to women of childbearing age in rural Nigeria.

## 2. Research Design

### 2.1. Sampling Design

We conducted our study in the Jada local government area (LGA) of Adamawa state in the northeastern region of Nigeria. The study was implemented in October 2016.

We employed the following two-stage sampling for women. First, we selected a total of 41 villages in 8 wards out of 11 in Jada LGA. Second, in each village, with a help of village heads, we created a census list of eligible women. A woman was eligible if she was aged between 15 and 35 and had never received a tetanus vaccine before. The list indicated whether she was pregnant or not. From the census list, we selected one woman from each household. If there was more than one eligible woman in a household, we prioritized and selected a pregnant woman if there were any. If there was more than one pregnant woman, we randomly selected one of them. If there were no pregnant women and more than one eligible non-pregnant woman, we randomly selected one non-pregnant woman. The census list identified 1747 eligible women from 1249 households in 41 villages. Selecting one eligible woman from each household, we sampled 1249 women in total.

**Figure 1:**
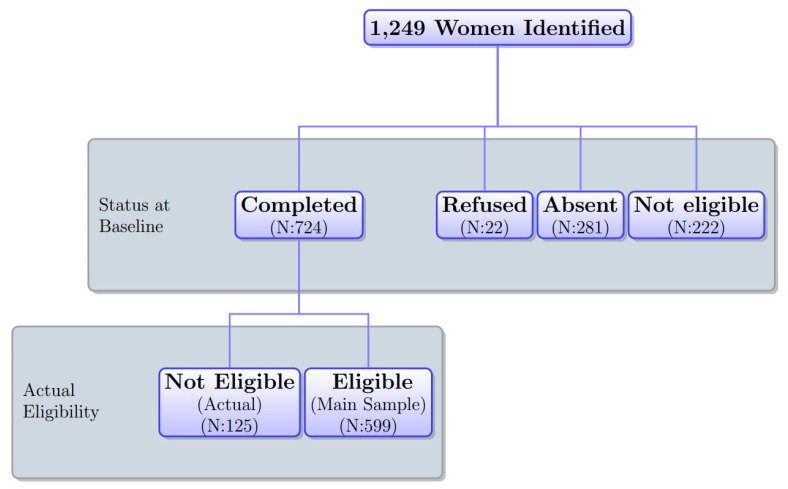
Sample. Notes: N is the number of observations.

Figure 1 depicts the distribution of women’s status in the sample at the time of the baseline survey. Of 1249 sampled women, 599 formed our analysis sample and the others were attrited due to refusal, absence, and ineligibility due to an inaccurate census list explained below. The sample attrition rate at the baseline was very high (52.0%). We address potential attrition bias in our analysis below.

### 2.2. Research Design and Procedure

#### 2.2.1. Research Design 

Respondents were randomly assigned to either the treatment or control group. Between the control and treatment groups, the task to be completed by women was different. The task for women in the control group was to answer a simple set of questions related to vaccination. On the other hand, the task among women in the treatment group was to receive the vaccination at their house, in addition to answering the same questions as the control group. To receive the vaccination, women in the treatment group were asked to submit the voucher. We explain the detailed procedure, as well as the interpretation of this design, below.

#### 2.2.2. Procedure

**Figure 2:**
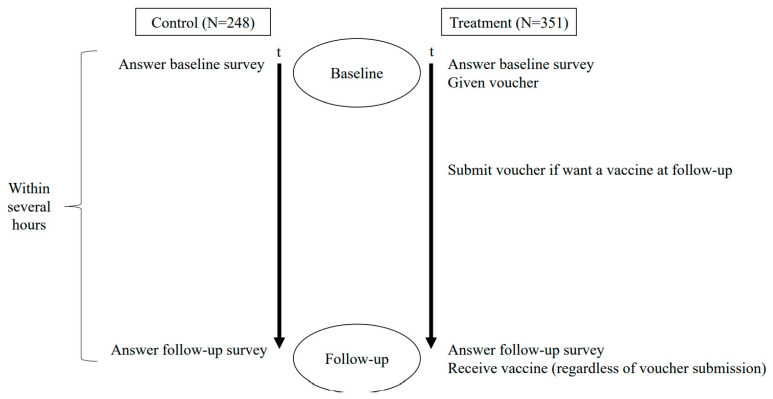
Timeline.

Figure 2 shows the timeline of the study. At the beginning of the baseline survey, interviewers asked women if they were willing to participate in the study at their house. One interviewer interviewed one participant at a time. They were orally informed by interviewers that the objective of the study was to understand barriers to health behaviors in general. Once respondents agreed to participate (22 refused), they orally provided consent and interviewers indicated the consent in the questionnaire form on behalf of respondents.

After obtaining consent, interviewers conducted the baseline survey with each participant to measure underlying attitudes, beliefs, and knowledge about vaccination and other health behaviors, as well as the women’s demographic and socioeconomic characteristics. The baseline survey took place in the morning at participants’ houses. It took about 15 min. In the baseline survey, respondents were informed by interviewers that a nurse would visit them to ask questions about vaccination several hours later in the afternoon. At the end of the baseline survey, interviewers also briefly explained the tetanus-toxoid vaccine to all respondents.

Our project hired 15 nurses as interviewers. We hired nurses because the follow-up survey involved vaccinating respondents at each respondent’s house. Respondents were not informed that interviewers were nurses at the baseline survey. Nurses confirmed the status of women’s tetanus-toxoid vaccination on the census list at the beginning of the baseline survey, finding 222 ineligible cases (Figure 1). During the baseline survey, additional 125 women were found to be ineligible due to either age or tetanus-vaccination status. The same nurses went back to the same household they visited for the baseline survey for a logistic reason; with no official address, nurses spent substantial time identifying women at the baseline. 

**Figure 3:**
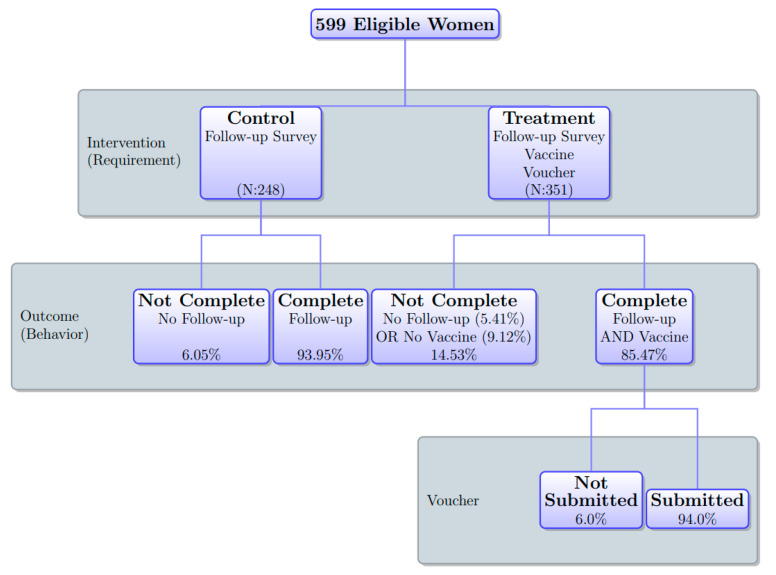
Research design and outcome. Notes: N is the number of observations. The percentage in the “Outcome” shows the percentage of women who fell into each category under each intervention arm (control or treatment), and the percentage in the “Voucher” shows the percentage of women who fell into each category among those who completed the requirement under the treatment arm.

Figure 3 presents the research design and outcome. The intervention took place at the end of the baseline survey. Respondents in the control group were informed that a nurse would visit them to ask some questions about vaccination in the follow-up survey at their house several hours after the baseline survey. Respondents in the treatment group were given the same information about the follow-up survey as women in the control group; in addition to that, they were also informed of the opportunity to receive a free tetanus vaccine at their house at the time of the follow-up survey. Interviewers provided each respondent in the treatment group with a voucher that was redeemable for a tetanus-toxoid vaccination. If they were willing to receive the vaccine at the time of the follow-up survey, they were instructed to submit the voucher to a member of the project who was stationed at the village head’s house before an interviewer visited them for the follow-up survey. They were informed that if they submitted the voucher, they could receive a free vaccine at their house at the same time as the follow-up survey. We used the village head’s house for women to submit the voucher because it was place known to all respondents. We did not use the health clinic for voucher submission to avoid any psychological factors related to health clinics influencing their decision about vaccination. Some respondents might have psychological barriers to visiting the house of the village head that might be correlated with barriers directly associated with vaccination. However, since they did not need to see the village head when submitting the voucher, such psychological barriers are unlikely to have significantly affected their submission decision.

Using villages as strata, we randomized the assignments of treatment status among 1249 women on the census list before interviewers visited the villages for the baseline survey. In each village, we randomly selected 45% of eligible women as the control group and the other 55% as the treatment group. We intentionally assigned more respondents to the treatment group because we planned to conduct the analysis of differential behaviors among treated women, depending on whether they submitted the voucher for the vaccine. The actual overall assignment ratio for the treatment among 599 women in the analysis sample was 58.6%. Thus, compared to the original sample, a few more women in the analysis sample were in the treatment group overall. We address this systematic attrition below.

Several hours after the baseline survey, interviewers (nurses) who conducted the baseline survey went back to the same respondents’ houses for the follow-up survey. The survey took no more than 10 min. The follow-up survey asked all the respondents why they thought that some people in general refuse to receive tetanus vaccines even if they are free. Respondents in the control group were additionally asked hypothetical questions as to whether they would have accepted the vaccine if offered for free, and if they would not have accepted it for free, how much money would have been sufficient for them to accept the vaccine offer.

Respondents in the treatment group were asked if they had submitted the voucher. The actual submission of the voucher was later reconciled with the receipt of the voucher at the village head’s house. In the analysis, we only refer to the actual submission of the voucher confirmed with the receipt but not the self-reported voucher submission. Then, regardless of the submission of the voucher, respondents were asked whether they would like to receive the vaccine right away. Thus, even if they did not submit the voucher, they had a chance to receive the vaccine if they orally accepted to do so. The offer to provide the free tetanus vaccine regardless of the voucher submission was a surprise to all the respondents in the treatment group. We used the voucher system and surprise offer of vaccine to evaluate differences in respondents’ willingness to pay (WTP) for the vaccine among respondents in the treatment arm. We explain more in the next subsection.

The follow-up survey data contain the following three measures: the completion of the follow-up survey, the submission of the voucher, and the vaccine take-up. The submission of the voucher and the vaccine take-up are applicable only for respondents in the treatment group.

### 2.3. Interpretation of Research Design

Our main outcome variable is whether respondents completed their task. The control task of women in the control group was to respond to the follow-up survey, and the treatment task of women in the treatment group was to respond to the follow-up survey and to receive the vaccine. The strict treatment task for women in the treatment group was to submit the voucher in addition to responding to the follow-up survey and receiving the vaccine. In this way, the definition of task completion was different for the control and treatment groups. We employed a similar unconventional design to elicit revealed preference in our previous study [23]. In that study, the condition under which women could receive cash incentives was differentiated: the condition in the control group was a clinic visit, while the condition in the treatment group was the uptake of a tetanus vaccine in addition to the clinic visit. This previous study evaluated the difference in accepting the conditions for receiving a cash reward between groups and revealed individuals’ willingness to give up money in order not to receive a vaccination (i.e., vaccine hesitancy). The research design of our current study builds on this previous work. We significantly improved the design by implementing the experiment at respondents’ houses instead of a health clinic to eliminate any potential hesitancy to clinic visits.

**Figure 4:**
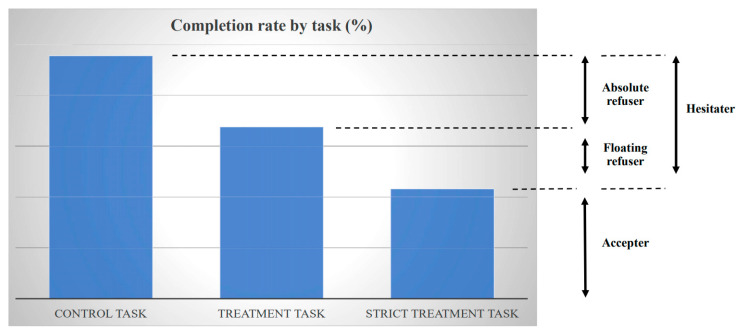
Interpretation of experiment. Notes: “Control Task” is the completion of the follow-up survey. “Treatment Task” is the completion of the follow-up survey and vaccination in the treatment group. “Strict Treatment Task” is the completion of the follow-up survey, vaccination, and the submission of the voucher in the treatment group. WTP < 0 among absolute refusers, and WTP ≅ 0 (or weakly positive) among floating refusers.

According to the voucher submission and the actual vaccine take-up among respondents in the treatment group, we categorized women into three types: (1) accepter, (2) floating refuser, and (3) absolute refuser, as illustrated in Figure 4. 

An accepter refers to a respondent who submitted the voucher and received the vaccine after responding to the follow-up survey, i.e., completing the strict treatment task. The submission of the voucher at the village head’s house revealed a high WTP for the vaccine because some opportunity costs were incurred in the voucher submission. Distinct from a conventional way of eliciting WTP through people’s self-reporting (stated preference), we captured the WTP from women’s actual behaviors (revealed preference). To elicit women’s WTP for the vaccine that is free of charge, we observed their actual behaviors sacrificing their time to receive the vaccine, in particular, reducing their time for work, household chore, rest, and so forth to spend time to travel to village head’s house (which involves no transportation costs). That is, women’s WTP for the vaccine was revealed through their willingness to pay their opportunity cost of receiving the vaccine by sacrificing their time. Although our design does not reveal monetary WTP, it captures a broad WTP for the vaccine. A floating refuser refers to a respondent who did not submit the voucher in advance but agreed to receive the vaccine at the respondent’s house at no cost after responding to the follow-up survey; the WTP for the vaccine was thus non-negative but low. In other words, if the vaccination were to cost even a small amount, either monetarily or non-monetarily, such as having to travel somewhere nearby, such as the village head’s house, floating refusers would not receive a vaccine. However, if the cost of receiving a vaccine were zero, they would receive the vaccine. Floating refusers were captured by the difference in the completion rates between the treatment task and the strict treatment task. The completion rate of the strict treatment task should be lower than that of the treatment task, because respondents needed to submit the voucher in addition to the treatment task. Finally, an absolute refuser refers to a respondent who did not submit the voucher and did not receive the vaccine after responding to the follow-up survey, even though accepting the surprise offer of the vaccine without submitting the voucher involved no cost. The WTP for the vaccine was strictly negative. Absolute refusers were captured by the difference in the completion rates between the control task and the treatment task. Vaccine hesitaters were a combination of absolute refusers and floating refusers, captured by the difference between the control task and the strict treatment task. Voucher submission was not randomized because our purpose was for respondents to reveal their WTP through their submission decision.

The difference between the completion of the control task and the treatment task/strict treatment task captured the prevalence of vaccine refusal/vaccine hesitancy under the following assumptions: first, the likelihood of responding to the follow-up survey was not affected differently by the treatment status—we formally test this assumption as described below; second, any factors other than vaccine hesitancy affected the completion of the follow-up survey in the same way for both the treatment and the control group.

### 2.4. Specification

To identify whether vaccine hesitancy and vaccine refusal are prevalent, we evaluated the effect of offering a vaccine on a respondent’s likelihood not to receive it. We estimated the effects of the treatment on the completion of the task with the following regression framework:

(1) $$Y_{ijk}=\alpha +\beta_{1}Treatment_{ijk}+\upsilon_{j}+\epsilon_{ijk}$$

where *Y_ij_* is a dummy variable that takes a value of 1 if a woman *i* in village *j* completes the task according to the treatment status (control task: responding to the follow-up survey; treatment task: responding to the follow-up survey and receiving the vaccine; and strict treatment task: the treatment task plus submitting the voucher) and *Treatment* is a treatment dummy that takes a value of 1 if a woman *i* is assigned to the treatment group. Since the treatment assignment was randomized at the level of individuals within villages (strata) and the assignment ratios varied across villages, we controlled for the village fixed effect *v_j_* to identify the treatment effect. Village fixed effects also controlled for village heterogeneity, including unobserved factors that affected attrition patterns. We clustered standard errors by village (41 villages in total) for conservative inference. We used ordinary least squares (OLS) regression for a simple interpretation of the prevalence of refusers. An alternative non-linear model would yield results that would be difficult to interpret because floating refusers are captured by the difference in the completion rates between the treatment task and the strict treatment task, as discussed above (Figure 4). Nonetheless, we also employed logistic regression to verify the robustness of our findings, finding qualitatively consistent results.

### 2.5. Robustness Checks

A potential threat to the identification from our estimates of treatment effects was the systematic attrition at the baseline survey, arguably driven by the interviewers, as discussed below. We conducted several robustness checks, including subsample analyses, fixed effect analysis to control for interviewer heterogeneity, and bound analysis, as detailed below.

## 3. Results 

### 3.1. Attrition

This subsection assesses the systematic attrition from the original sample identified on the census list (1249 women) to the analysis sample who were eligible and responded to the baseline survey (599).

We tested whether the attrition rate in the original sample was significantly different in relation to the treatment status by regressing the attrition status at the baseline on the treatment status (Table 1 panel A). The results showed that the overall attrition—the proportion of women who were not eligible or did not complete the baseline survey—was 6.9 percentage points lower in the treatment group than the control group (column 1). There were three reasons for attrition: non-eligibility, absence, and refusal (Figure 1). The results show that this systematic attrition was mainly caused by absence (281): whereas the proportions of ineligible women and of women who refused to participate in the baseline survey were not significantly different between the treatment and control groups (columns 2 and 4), women being absent at the time of the baseline survey was less common by 4.4 percentage points in the treatment group than the control group (column 3).

To address the systematic attrition potentially driven by interviewers’ systematic selection, we conducted two analyses. First, the systematic attrition patterns might have varied among interviewers if the degree of their systematic selection varied. Repeating the analysis for each interviewer showed that the treatment status was negatively and significantly correlated with attrition for 5 out of 15 interviewers. Excluding respondents who were interviewed by these five interviewers, the overall attrition was no longer correlated with the treatment status (Table 1 Panel B column 1), although the absence rate was still lower among women in the treatment arm (column 3).

Second, systematic attrition patterns might have varied across villages. This was because each interviewer went to a certain village assigned per day, and thus the attrition by the interviewer might have systematically varied across villages. Some village characteristics might also have affected the degree of interviewers’ systematic selection through their differential efforts to find respondents. Repeating the analysis for each village showed that the attrition rate was negatively and significantly correlated with the treatment status in 5 out of 41 villages. Excluding respondents who were from these five villages, the attrition was no longer correlated with the treatment status for any measures (Table 1 Panel C). In particular, the systematic attrition due to absence that remained in the subsample, excluding the five interviewers who selectively conducted interviews, disappeared. Thus, there was no evidence for systematic attrition in this subsample.

### 3.2. Balancing Tests

Table 2 presents the summary statistics of baseline covariates among 599 women in the analysis sample according to treatment status. On average, respondents were 22 years old. Over half of the respondents had no education or did not complete primary school, around 35% had primary-school education, and around 14% had secondary-school education or higher. Sixty-five percent had been married (most of them were currently married). More than half (58%) of respondents belonged to the Chamba ethnic group and 25% were Fulani. The majority of respondents, almost 80%, were Muslims, as almost all Fulani and half of Chamba are Muslims.

On average, women delivered 2 babies, and 1.5 of them were alive. Thirteen percent of women were pregnant at the time of the study. On average, it took 7.7 min for respondents to walk from their house to the village head’s house, where women in the treatment arm could submit the voucher for the vaccination. Most roofs were finished in materials such as metal. About 47% of respondents had paid work. They earned about one-tenth of household earnings on average. The average household monthly income was 20,000 naira (1 USD = 311 naira, October 2016). Almost half of respondents had received any injection-type vaccines, including ones not for tetanus, before.

Table 2 suggests that our randomization performed well, except for the time taken to walk from women’s houses to the village head’s house and pregnancy status. We controlled for the baseline covariates reported in Table 2 in the regression analysis below.

### 3.3. Follow-Up Survey Completion by Treatment

One of the assumptions required to measure the prevalence of vaccine refusal (vaccine hesitancy) by comparing the control task and the treatment task (strict treatment task) was that the completion of the follow-up survey was not affected differently by the treatment status. This subsection assesses the plausibility of this assumption.

Of the 599 women in the analysis sample, when they were asked at the baseline survey whether they intended to participate in the follow-up survey later, 95.2% answered yes. Interviewers tried to return to all the women, including those who had indicated that they had no intention of participating in the follow-up survey; 94.3% of the women actually participated in the follow-up survey (all of them completed it), while 2.7% refused and 3.0% were absent.

We examined whether these follow-up survey statuses—intention to participate, completion, refusal, and absence—were correlated with the treatment status. The completion status of the follow-up survey can be correlated with the treatment status. For example, women in the treatment group might have felt social pressure to accept the vaccine when they agreed to participate in the follow-up survey, even though they actually did not want to receive the vaccine. Because we informed the treated women that a nurse would visit them for the follow-up survey and vaccination, they might have been absent from the follow-up survey to avoid explicitly refusing the vaccine or might have actually refused the follow-up survey. In contrast, women in the control group might have found it unnecessary to avoid completing the follow-up survey, which was independent from the vaccination. The results showed that the treatment status was not associated with any of these follow-up survey statuses (Table 3 Panel A). Since the systematic attrition at the baseline survey was found to be driven by the systematic selection made by interviewers, we additionally controlled for interviewer fixed effects to check the robustness of our findings, finding similar results (Table 3 Panel B). Thus, the completion of the follow-up survey was not affected differently by the treatment status, and, distinct from the attrition at the baseline survey, the additional attrition at the follow-up survey was not systematically related to the treatment status either.

### 3.4. Vaccine Hesitancy

**Figure 5:**
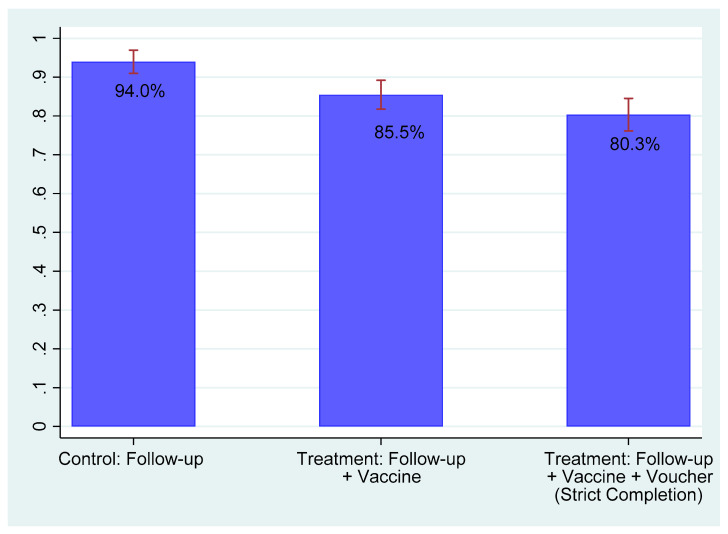
Completion rate by treatment status. Notes: “Completion” in the control group means the completion of the follow-up survey (control task). “Completion” in the treatment group is the completion of the follow-up survey and vaccination (treatment task). “Strict Completion” in the treatment group is the completion of the follow-up survey, vaccination, and the submission of the voucher (strict treatment task). The confidence interval is at a 95% level.

Figure 5 presents the completion rate according to the treatment status. As discussed above, the task to be completed was different according to the treatment status. Among women in the control arm, the control task was responding to the follow-up survey. Among women in the treatment arm, the treatment task additionally involved receiving the vaccination right after the follow-up survey (i.e., control task plus the receipt of the vaccination) and the strict treatment task further involved submitting the voucher prior to the follow-up survey (i.e., treatment task plus the submission of the voucher). Over 90% of women in the control group completed the control task. About 85% of women in the treatment group completed the treatment task. About 80% of respondents in the treatment group completed the strict treatment task.

Table 4 presents the estimates for the effect of the treatment on the completion of the tasks. The results show that the prevalence of both vaccine hesitancy and absolute vaccine refusal was significant. The results were similar with and without the covariates listed in Table 2 plus controlling for age squared (panel A). Controlling for covariates, the estimated prevalence of vaccine hesitancy, which is the difference between the completion rates of the control task and of the strict treatment task, was 13.1% (Table 4 Panel A column 4), and the estimated prevalence of absolute vaccine refusal, which was the difference between the completion rates of the control task and of the treatment task, was 7.0% (Table 4 Panel A column 2). The difference between these two estimates, 6.1%, was the difference between the completion rates of the treatment task and the strict treatment task, which, as illustrated in Figure 4, captured the prevalence of floating refusal. This estimated difference of 6.1 percentage points was not statistically different from 0 at conventional levels, however. Some respondents in the treatment arm who did not submit the voucher might have felt pressured to receive the vaccine when they were given a surprise offer of the vaccine during the follow-up survey. We purposely instructed nurses not to enforce vaccination. Some respondents might nevertheless have felt pressured. Then, the estimate of the prevalence of floating refusal would be biased upward.

Overall, we find that the prevalence of vaccine hesitancy is about 13%. The vaccine hesitaters were almost equally divided between absolute refusers and floating refusers. We asked for reasons for not receiving the vaccine among women in the treatment group. Common self-reported reasons were that the vaccine is painful (73.3%), the vaccine is not necessary (56.7%), the vaccine is harmful (30.0%), and the vaccine is not effective (26.7%) (Table 5). Interpreting these results requires caution because the number of observations is small due to missing values. Although the reasons respondents thought that the vaccine is not necessary is unclear, these results provide suggestive evidence that physical pain might be as important as a barrier to vaccination as distrust or negative belief.

### 3.5. Robustness Check

In this subsection, we describe a battery of robustness checks, showing that attrition bias was unlikely to affect our estimation results.

First, we estimated treatment effects in the two subsamples for which we found limited systematic attrition above: one excluding five specific interviewers and another excluding five villages with significant selection (Table 1 Panels B and C). The results showed that in both subsamples, the estimated treatment effects were similar to the original results, regardless of whether the covariates were controlled for (Table 4 panels B and C).

Second, we controlled for interviewer fixed effects, in addition to village fixed effects, to control for interviewer heterogeneity including unobserved factors that would affect the interviewers’ systematic selection. The results for the whole sample reported in Table 6 Panel A are very similar to the original results in Table 4 Panel A. The results for the two subsamples with limited systematic attrition reported in Table 6 Panels B and C are also similar to those in Table 4 Panels B and C.

Third, we conducted two analyses to assess the robustness of the treatment effect estimates to potential attrition bias. For the 125 women who were found to be ineligible during the baseline survey (Figure 1), we still conducted the follow-up survey. Adding these women to the analysis sample increased the sample from 599 to 724. The first analysis re-estimated the treatment effects in this expanded analysis sample using the actual outcome measures for these ineligible women. The second analysis bounded the estimates of the treatment effects for possible attrition bias by imputing outcome values for the remaining 525 attritors of the 1249 women on the census list. We considered the scenarios that would reduce treatment effect estimates in magnitude: for the attrited respondents in the control group, we imputed high values, which were the observed control mean plus 0.1, 0.25, and 0.5 standard deviations of the control distribution, and for the attrited respondents in the treatment group, we imputed low values, which were the observed treatment mean minus 0.1, 0.25, and 0.5 standard deviations of the treatment distribution. Since our outcomes were dummy variables, these calculations were based on imputations of zeros and ones to actual individuals in the data, as conducted by Kling and Liebman (2004). The results are reported in Table 7. The estimates of the treatment effects on completion and strict completion in the expanded sample including ineligible women with outcome measures (column 3) are similar to the original estimates reported in panel A of Table 4 (column 1). The re-estimated treatment effects for completion and strict completion are robust to 0.1 and 0.25 standard deviations, respectively (columns 4–6). Compared to previous works conducting the same sensitivity analysis, the degree of robustness found is similar to Blattman et al. (2014) [24] and much stronger than Karlan and Valdivia (2011) [25] and Drexler, Fischer, and Schoar (2014) [26].

### 3.6. Correlation between Belief and Behavior

Conventional wisdom is often formed from observational studies using subjective measurements. In this subsection, we compare subjective beliefs regarding vaccination with the behavioral evidence of the prevalence of vaccine hesitancy. We focus on women in the treatment group in this subsection because only they had the opportunity to receive the vaccine in our study.

Table 8 examines the correlations between the subjective beliefs about vaccination and actual vaccine take-up. Specifically, we regress the dummy for the completion of each task, treatment task, or strict treatment task, for each of the 11 belief measures measured in the baseline survey. This analysis does not have a causal interpretation. Although some belief measures are significantly correlated with the completion of each task, they are not robust to multiple hypothesis testing.

Table 9 shows other suggestive but weak evidence for the correlation between the vaccine take-up and the subjective beliefs about vaccines, from open-ended questions regarding concerns about vaccines asked to all respondents. For each of the 17 concerns about vaccines, we compared the completion of the treatment task among women in the treatment group (column 1 vs. 2). We found that more respondents who did not complete the treatment task stated concerns about vaccines, such as being afraid of the vaccine and concerns about headache and fever, though none of the differences were statistically significant. Similar patterns were found for the strict treatment task (column 3 vs. 4).

Overall, the association of subjective beliefs about vaccination and actual vaccination behaviors was limited: weak correlations were found only for a small number of subjective measures. That is, women’s beliefs only weakly corresponded to their behaviors. 

## 4. Discussion and Conclusions

This study behaviorally examined the prevalence of vaccine hesitancy. We conducted a randomized controlled trial in rural northern Nigeria among women of childbearing age with no previous experience of tetanus vaccination. We found that the prevalence of vaccine hesitancy is about 13%. In contrast, subjective measures of vaccine beliefs commonly used in observational studies only weakly corresponded to behaviors. This result suggests a limitation to an observational approach, which is commonly found in the emerging literature on vaccine hesitancy [22], and emphasizes the importance of an experimental approach to measure the prevalence of vaccine hesitancy. 

Our research design distinguished two types of vaccine hesitaters: absolute refusers, who have a negative WTP for the vaccine, and floating refusers, with almost-zero WTP. Effective policies to address each type of vaccine hesitancy are different. Floating refusers can easily agree to receive vaccines if the costs of doing so are low enough. A simple intervention, such as a door-to-door vaccination campaign, is likely to be effective for these people. Some door-to-door vaccination campaigns such as polio vaccination campaign in Nigeria [15] induced high immunization rate. In contrast, absolute refusers actively refuse to receive a vaccine, and the door-to-door campaign is less likely to change their vaccine behavior. To induce behavioral change among absolute refusers from refusal to the acceptance of vaccination, policies first need to lower the barriers directly associated with vaccines, such as misperception and distrust of vaccines. Designing effective interventions to this end is a major research topic in the literature [12,27]. Our finding of the similar prevalence of absolute refusers and floating refusers suggests that two distinct sets of policies for each target group are equally needed.

### Limitations

Our study has several limitations. First, our choice of the study area, Jada LGA in Adamawa state in Nigeria, limits the external validity of our findings. Second, our study in rural areas did not allow us to capture any differential behaviors in terms of vaccine hesitancy between rural and urban areas. Third, although we conducted an extensive robustness check of our analyses, they could be vulnerable to potential bias caused by the differential attrition. Fourth, although we were extremely cautious, the use of nurses as surveyors may have imposed some pressure on respondents. Fifth, this paper exclusively focused on women’s behaviors around tetanus vaccination, which limits the external validity to other types of vaccination. Sixth, vaccination decisions for women might have been made by household heads. This study is unable to distinguish vaccine hesitancy among women and among decision makers (household heads) for women. Seventh, our experiment took place at respondents’ houses to evaluate their willingness to accept vaccination. This setting is rather unique in comparison to the standard immunization services usually offered at health facilities; thus, it limits the external validity of our findings. However, our research design was employed to reveal the vaccine hesitancy in an innovative way. Eighth, measuring willingness to pay through voucher submission is narrowly scoped. It is stressed that voucher submission is only one aspect of showing the willingness to pay for vaccination. Ninth, our study focused only on vaccine hesitancy among various reasons for non-vaccination. Lastly, it is beyond the scope of the paper to identify reasons for vaccine hesitancy. 

## Figures and Tables

**Table 1:** Attrition at baseline survey.

	Overall Attrition	Ineligible	Absent	Refused
Panel A: Whole sample				
	(1)	(2)	(3)	(4)
Treatment status (=1 if Treatment)	−0.069 **	−0.027	−0.044 *	0.002
	(0.027)	(0.025)	(0.023)	(0.007)
N	1249	1249	1249	1249
r2	0.005	0.001	0.003	0.000
Control mean of dependent variable	0.559	0.295	0.247	0.016
Panel B: Exclude 5 Interviewers (out of 15)				
	(1)	(2)	(3)	(4)
Treatment status (=1 if Treatment)	−0.028	0.017	−0.052 *	0.006
	(0.032)	(0.030)	(0.029)	(0.010)
N	855	855	855	855
r2	0.001	0.000	0.004	0.001
Control mean of dependent variable	0.585	0.292	0.276	0.018
Panel C: Exclude 5 villages (out of 41)				
	(1)	(2)	(3)	(4)
Treatment status (=1 if Treatment)	−0.022	−0.000	−0.030	0.008
	(0.029)	(0.026)	(0.025)	(0.008)
N	1069	1069	1069	1069
r2	0.001	0.000	0.001	0.001
Control mean of dependent variable	0.528	0.270	0.246	0.012
Village fixed effects	X	X	X	X

Notes: All the specifications are with village fixed effects. Robust standard errors clustered by villages (41 villages) are shown in parentheses. * significant at 10%; ** significant at 5%, *** significant at 1%

**Table 2:** Baseline balance.

	Control	Treatment	Difference
	(1)	(2)	(3)
Age	21.835	21.955	0.209
Education = None	0.379	0.358	−0.015
Education = Below Primary	0.157	0.129	−0.031
Education = Primary	0.335	0.358	0.016
Education = Secondary or higher	0.129	0.155	0.030
Single	0.355	0.334	−0.027
Ethnicity = Chamba	0.573	0.586	0.027
Ethnicity = Fulani	0.254	0.254	−0.009
Ethnicity = Other	0.173	0.16	−0.018
Muslim	0.766	0.8	0.029
Number of babies delivered	2.020	1.945	−0.058
Pregnant	0.113	0.145	0.044 *
Minutes to village head house	8.974	6.753	−2.264 **
Number of household members	7.976	8.542	0.579
Roof material (natural)	0.270	0.236	−0.034
Roof material (rudimentary)	0.084	0.063	−0.020
Roof material (finished)	0.637	0.689	0.052 *
Has paid work	0.435	0.490	0.042
Ever received injection-type vaccine	0.494	0.491	−0.006

Notes: The total number of observations varies from 566 to 599, depending on the number of missing values. The treatment-control mean differences are with village fixed effects, with clustered standard errors (village-level). Roof material indicates the type of material used for the roof, which captures the wealth of the household. * significant at 10%; ** significant at 5%, *** significant at 1%.

**Table 3:** Status of follow-up survey.

	Intention to Participate	Completed	Absent	Refused
Panel A: Village Fixed Effects				
	(1)	(2)	(3)	(4)
Treatment	−0.026	0.006	−0.005	−0.002
	(0.023)	(0.022)	(0.016)	(0.011)
N	599	599	599	599
r2	0.004	0.000	0.000	0.000
Control mean of dependent variable	0.974	0.939	0.030	0.030
Fixed effects (village)	X	X	X	X
Panel B: Village and Interviewer Fixed Effects				
	(1)	(2)	(3)	(4)
Treatment	−0.016	0.011	−0.007	−0.004
	(0.022)	(0.022)	(0.015)	(0.012)
N	599	599	599	599
r2	0.039	0.028	0.032	0.034
Control mean of dependent variable	0.974	0.939	0.030	0.030
Fixed effects (village and interviewer)	X	X	X	X

Notes: The sample is 599 women in the analysis sample. The estimates are with village fixed effects in panel A and village and interviewer fixed effects in panel B. Robust standard errors clustered by villages (41 villages) are shown in parentheses. Covariates are age, age squared, education level (dummies), marital status, ethnicity (dummies), religion, the number of babies delivered, pregnancy status, minutes to the village head’s house, total number of household members, roof material (dummies), whether a respondent has paid work, and whether a respondent has ever received an injection-type vaccination.

**Table 4:** Main results.

Dependent Variable:	Completion		Strict Completion	
Definition of “Completion”	Follow-Up (Control) vs. Follow-Up + Vaccination (Treatment)		Follow-Up (Control) vs. Follow-Up + Vaccination + Voucher Submission (Treatment)	
Panel A: Whole sample				
	(1)	(2)	(3)	(4)
Treatment status (=1 if Treatment)	−0.078 **	−0.070 **	−0.128 ***	−0.131 ***
	(0.029)	(0.029)	(0.033)	(0.036)
N	599	557	599	557
r2	0.017	0.061	0.038	0.082
Control mean of dependent variable	0.939	0.939	0.939	0.939
Panel B: Exclude 5 Interviewers (out of 15)				
	(1)	(2)	(3)	(4)
Treatment Status (=1 if Treatment)	−0.068 **	−0.060 *	−0.122 ***	−0.129 ***
	(0.026)	(0.030)	(0.025)	(0.031)
N	368	331	368	331
r2	0.015	0.087	0.038	0.110
Control mean of dependent variable	0.946	0.946	0.946	0.946
Panel C: Exclude 5 villages (out of 41)				
	(1)	(2)	(3)	(4)
Treatment status (=1 if Treatment)	−0.082 **	−0.075 **	−0.129 ***	−0.134 ***
	(0.032)	(0.031)	(0.036)	(0.039)
N	517	476	517	476
r2	0.022	0.073	0.045	0.085
Control mean of dependent variable	0.947	0.947	0.947	0.947
Covariates		X		X
Fixed effects (village)	X	X	X	X

Notes: The sample is 599 women in the analysis sample. Some observations with missing values in covariates are dropped in columns (2) and (4). Robust standard errors clustered by villages (41 villages) are shown in parentheses. Covariates are age, age squared, education level (dummies), marital status, ethnicity (dummies), religion, the number of babies delivered, pregnancy status, minutes to the village head’s house, total number of household members, roof material (dummies), whether a respondent has paid work, and whether a respondent has ever received an injection-type vaccination. * significant at 10%; ** significant at 5%; and *** significant at 1%.

**Table 5:** Reasons for not receiving vaccine.

	%
Vaccine is harmful	30.00
Vaccine is painful	73.33
Vaccine is not necessary	56.67
Vaccine is not effective	26.67
I want to avoid the nurse	16.67
Husband does not allow it	10.00
I am not interested in the vaccine	6.67
I am afraid of the vaccine	6.67
Other reasons	16.67

Notes: The sample is 35 women in the treatment group who refused to receive the vaccine.

**Table 6:** Main results with interviewer fixed effects.

Dependent Variable:	Completion		Strict Completion	
Definition of “Completion”	Follow-Up (Control) vs. Follow-Up + Vaccination (Treatment)		Follow-Up (Control) vs. Follow-Up + Vaccination + Voucher Submission (Treatment)	
Panel A: Whole sample				
	(1)	(2)	(3)	(4)
Treatment status (=1 if Treatment)	−0.072 **	−0.066 **	−0.125 ***	−0.127 ***
	(0.030)	(0.032)	(0.035)	(0.038)
N	599	553	599	553
r2	0.142	0.166	0.134	0.171
Control mean of dependent variable	0.939	0.939	0.939	0.939
Panel B: Exclude 5 Interviewers (out of 15)				
	(1)	(2)	(3)	(4)
Treatment status (=1 if Treatment)	−0.058 **	−0.041	−0.113 ***	−0.107 ***
	(0.027)	(0.033)	(0.027)	(0.031)
N	368	328	368	328
r2	0.136	0.181	0.131	0.206
Control mean of dependent variable	0.946	0.946	0.946	0.946
Panel C: Exclude 5 villages (out of 41)				
	(1)	(2)	(3)	(4)
Treatment status (=1 if Treatment)	−0.067 **	−0.063 *	−0.119 ***	−0.121 ***
	(0.032)	(0.033)	(0.036)	(0.039)
N	517	472	517	472
r2	0.163	0.189	0.141	0.171
Control mean of dependent variable	0.947	0.947	0.947	0.947
Covariates		X		X
Fixed effects (village and interviewer)	X	X	X	X

Notes: The sample is 599 women in the analysis sample. Some observations with missing values in covariates are dropped. The estimates are with village and interviewer fixed effects. Robust standard errors clustered by villages (41 villages) are shown in parentheses. Covariates are age, age squared, education level (dummies), marital status, ethnicity (dummies), religion, the number of babies delivered, pregnancy status, minutes to the village head’s house, total number of household members, roof material (dummies), whether a respondent has paid work, and whether a respondent has ever received an injection-type vaccination. * significant at 10%; ** significant at 5%; and *** significant at 1%.

**Table 7:** Sensitivity analysis.

	Unadjusted Estimates			Bound Estimates		
	Analysis Sample		Expanded Sample	0.10 SD	0.25 SD	0.50 SD
	(1)	(2)	(3)	(4)	(5)	(6)
Completion	−0.078 **	−0.085 **	−0.082 **	−0.050 **	−0.027	0.025
	(0.029)	(0.031)	(0.027)	(0.020)	(0.022)	(0.024)
Strict completion	−0.128 ***	−0.136 ***	−0.140 ***	−0.123 ***	−0.075 ***	−0.033
	(0.033)	(0.035)	(0.029)	(0.022)	(0.020)	(0.022)
Fixed effects (village)	X					
N	599	599	724	1249	1249	1249

Notes: The sample is 599 women in the analysis sample in columns (1) and (2), 724 women in the expanded analysis sample including ineligible women with outcome measures in column (3), and 1249 women on the census list in columns (4)–(6). Column (1) replicates the results reported in columns (1) and (3) in panel A of Table 4. Village fixed effects are not controlled for in columns (2)–(6). Covariates are not controlled for. For the bound analysis (columns 4–6), we consider the scenarios that would reduce treatment effect estimates in magnitude: for the attrited respondents in the control group, we impute high values, which are the observed control mean plus 0.1, 0.25, and 0.5 standard deviations of the control distribution, and for the attrited respondents in the treatment group, we impute low values, which are the observed treatment mean minus 0.1, 0.25, and 0.5 standard deviations of the treatment distribution. Following Kling and Liebman (2004), these calculations are based on imputations of zeros and ones to actual individuals in the data. Robust standard errors clustered by villages are shown in parentheses. * significant at 10%; ** significant at 5%; and *** significant at 1%.

**Table 8:** Beliefs and vaccine take-up among treated women.

Independent Variable:	Vaccine Causes HIV	Vaccine Protects Me	Vaccine Needle Is Scary	Vaccine Causes Fever/Head Ache	Vaccine Gives Disease	Vaccine Helps Me Stay Healthy	Vaccine Cures Disease	My Religion Is against the Vaccine	I Am against the Vaccine	I Do Not Care about the Vaccine	I Want to Get Vaccine When I Have a Chance
Panel A: Completion											
Dependent variable				Completion (Follow-up + Vaccination)							
	(1)	(2)	(3)	(4)	(5)	(6)	(7)	(8)	(9)	(10)	(11)
Coefficients	0.005	−0.086	−0.102	−0.158 **	−0.076	−0.034	−0.008	−0.057	−0.107	−0.103 *	0.174 **
	(0.130)	(0.100)	(0.071)	(0.077)	(0.069)	(0.079)	(0.073)	(0.064)	(0.069)	(0.059)	(0.069)
N	325	325	325	325	325	325	325	325	325	325	325
r2	0.246	0.249	0.254	0.260	0.250	0.247	0.246	0.248	0.256	0.258	0.260
Romano–Wolf stepdown *p*-values	0.970	0.901	0.703	0.347	0.822	0.951	0.970	0.901	0.614	0.356	0.208
Mean of dependent variables under x = 0	0.889	0.898	0.903	0.896	0.893	0.919	0.898	0.885	0.897	0.905	0.897
Panel B: Strict Completion											
Dependent variable			Strict Completion (Follow-up + Vaccination + Voucher submission)								
	(1)	(2)	(3)	(4)	(5)	(6)	(7)	(8)	(9)	(10)	(11)
Coefficients	0.009	−0.068	−0.044	−0.243 **	−0.019	−0.129	0.057	−0.011	−0.102	−0.135 **	0.164 **
	(0.147)	(0.089)	(0.083)	(0.093)	(0.071)	(0.083)	(0.068)	(0.069)	(0.081)	(0.061)	(0.073)
N	325	325	325	325	325	325	325	325	325	325	325
r2	0.254	0.256	0.256	0.283	0.255	0.266	0.256	0.254	0.262	0.273	0.266
Romano-Wolf stepdown *p*-values	0.980	0.941	0.970	0.158	0.970	0.465	0.941	0.980	0.802	0.129	0.287
Mean of dependent variables under x = 0	0.857	0.871	0.872	0.869	0.863	0.900	0.868	0.854	0.866	0.879	0.867
Mean of independent variables	0.040	0.184	0.137	0.088	0.145	0.297	0.214	0.132	0.192	0.351	0.160
Covariates	X	X	X	X	X	X	X	X	X	X	X
Fixed effects (village and interviewer)	X	X	X	X	X	X	X	X	X	X	X

Notes: The sample is 325 women in the treatment group. We regress the task completion on each belief measure separately. In both panels A and B, each column shows the estimated coefficient of each belief measure. The estimates are with village and interviewer fixed effects. Robust standard errors clustered by villages (41 villages) are presented in parentheses. Covariates are age, age squared, education level (dummies), marital status, ethnicity (dummies), religion, the number of babies delivered, pregnancy status, minutes to the village head’s house, total number of household members, roof material (dummies), whether a respondent has paid work, and whether a respondent has ever received an injection-type vaccination. “Mean of dependent variables under x = 0” shows the mean of dependent variables when the independent variables (x) take a value of 0. * significant at 10%; ** significant at 5%, *** significant at 1%.

**Table 9:** Concerns about receiving vaccines among treated women.

What Are the Concerns When Receiving Vaccine? (%)				
	Did Not Receive Vaccine	Received Vaccine	Did Not Submit Voucher or Did Not Receive Vaccine	Submitted Voucher and Received Vaccine
	(1)	(2)	(3)	(4)
I am afraid of the vaccine	10.42	7.69	15.38	6.32
Headache	2.08	0.35	1.54	0.37
Fever	6.25	4.20	4.62	4.46
Swelling	2.08	1.40	3.08	1.12
Sick	0.00	1.75	0.00	1.86
Painful	16.67	19.93	16.92	20.07
Afraid of needles	14.58	13.64	12.31	14.13
Vaccine is harmful	2.08	1.05	1.54	1.12
Vaccine makes women infertile	2.08	0.70	1.54	0.74
Distance to clinic	2.08	2.10	1.54	2.23
No vaccine at clinic	0.00	0.70	0.00	0.74
Smell of clinic	0.00	0.35	0.00	0.37
Long waiting time	0.00	0.35	0.00	0.37
Husband does not allow it	0.00	0.35	0.00	0.37
No knowledge about the vaccine	2.08	3.15	3.08	2.97
Do not know where to get the vaccine	0.00	0.35	0.00	0.37
No concern	39.58	41.96	38.46	42.38
Number of observations	48	286	65	269

Notes: The sample is 351 women in the treatment group with information in open-ended questions.

## Data Availability

The data that support the findings of this study are available from the corresponding author, R.S., upon reasonable request.

## Registration

The trial was registered at AEA RCT registry (ID: AEARCTR−0003563).

## References

[1] Ehreth J. (2003). The global value of vaccination. Vaccine.

[2] Kurosky S., Keith K., Davis L., Girishanthy K. (2016). Completion and compliance of childhood vaccinations in the United States. Vaccine.

[3] WHO (2017). Immunization Coverage.

[4] WHO (2017). Tetanus vaccines: WHO Position Paper. Wkly. Epidemiol. Rec..

[5] WHO (2013). Weekly Epidemiological Record.

[6] Thysen S.M., Byberg S., Pedersen M., Rodrigues A., Ravn H., Martins C., Benn C.S., Aaby P., Fisker A.B. (2014). BCG coverage and barriers to BCG vaccination in Guinea-Bissau: An observational study. BMC Public Health.

[7] Orimadegun A.E., Akinlolu A.A., Olusegun O.A. (2014). Adolescent girls’ understanding of tetanus infection and prevention: Implications for the disease control in western Nigeria. Front. Public Health.

[8] Jheeta M., James N. (2008). Childhood vaccination in Africa and Asia: The effects of parents’ knowledge and attitudes. Bull. World Health Organ..

[9] Steele F., Diamond I., Amin S. (1996). Immunization Uptake in Rural Bangladesh: A Multilevel Analysis. J. R. Stat. Soc. Ser. A Stat. Soc..

[10] Rainey J.J., Watkins M., Ryman T.K., Sandhu P., Bo A., Banerjee K. (2011). Reasons related to non-vaccination and under-vaccination of children in low and middle income countries: Findings from a systematic review of the published literature, 1999–2009. Vaccine.

[11] MacDonald N.E. (2015). Vaccine hesitancy: Definition, scope and determinants. Vaccine.

[12] Dubé E., Caroline L., Guay M., Bramadat P., Roy R., Bettinger J.A. (2013). Vaccine hesitancy: An overview. Hum. Vaccines Immunother..

[13] Smith T.C. (2017). Vaccine Rejection and Hesitancy: A Review and Call to Action. Open Forum Infect. Dis..

[14] The Lancet Child & Adolescent Health (2019). Vaccine hesitancy: A generation at risk. Lancet.

[15] Jegede A.S. (2007). What Led to the Nigerian Boycott of the Polio Vaccination Campaign?. PLoS Med..

[16] Centers for Disease Control and Prevention (2005). Progress toward interruption of wild poliovirus transmission—Worldwide, January 2004–March 2005. MMWR Morb. Mortal. Wkly. Rep..

[17] Kaufmann J.R., Feldbaum H. (2009). Diplomacy and the polio immunization boycott in Northern Nigeria. Health Aff..

[18] UNICEF (2001). Combatting Antivaccination Rumors: Lessons Learned from Case Studies in East Africa.

[19] UNICEF (2010). Maternal and Neonatal Tetanus Elimination Initiative.

[20] Feldman-Savelsberg P., Ndonko F.T., Schmidt-Ehry B. (2000). Sterilizing Vaccines or the Politics of the Womb: Retrospective Study of a Rumor in Cameroon. Med. Anthr. Q..

[21] Larson H.J., Jarrett C., Eckersberger E., Smith D.M.D., Paterson P. (2014). Understanding Vaccine Hesitancy around Vaccines and Vaccination from a Global Perspective: A Systematic Review of Published Literature, 2007–2012. Vaccine.

[22] Larson H.J., Jarrett C., Schulz W.S., Chaudhuri M., Zhou Y., Dubé E., Schuster M., MacDonald N.E., Wilson R. (2015). Measuring vaccine hesitancy: The development of a survey tool. Vaccine.

[23] Sato R., Takasaki Y. (2019). Psychic vs. Economic Barriers to Vaccine Take-up: Evidence from a Field Experiment in Nigeria. World Bank Econ. Rev..

[24] Blattman C., Fiala N., Martinez S. (2014). Generating skilled selfemployment in developing countries: Experimental evidence from Uganda. Q. J. Econ..

[25] Karlan D., Valdivia M. (2011). Teaching Entrepreneurship: Impact of Business Training on Microfinance Clients and Institutions. Rev. Econ. Stat..

[26] Drexler A., Fischer G., Schoar A. (2014). Keeping it simple: Financial literacy and rules of thumb. Am. Econ. J. Appl. Econ..

[27] Jarrett C., Wilson R., O’Leary M., Eckersberger E., Larson H.J. (2015). Strategies for addressing vaccine hesitancy–A systematic review. Vaccine.

